# Mindfulness meditation and explicit and implicit indicators of personality and self-concept changes

**DOI:** 10.3389/fpsyg.2015.00044

**Published:** 2015-01-29

**Authors:** Cristiano Crescentini, Viviana Capurso

**Affiliations:** ^1^Department of Human Sciences, University of UdineUdine, Italy; ^2^Department of Psychology, University of Rome La SapienzaRome, Italy

**Keywords:** mindfulness meditation, personality inventory, character, self-concept, explicit-attitude, implicit-attitude

## Abstract

The scientific interest on mindfulness meditation (MM) has significantly increased in the last two decades probably because of the positive health effects that this practice exerts in a great variety of clinical and non-clinical conditions. Despite attention regulation, emotional regulation, and body awareness have been argued to be critical mechanisms through which MM improves well-being, much less is known on the effects of this practice on personality. Here we review the current state of knowledge about the role of MM in promoting changes in practitioners’ personality profiles and self-concepts. We first focus on studies that investigated the relations between mindfulness and personality using well-known self-report inventories such as the Five-Factor model of personality traits and the Temperament and Character Inventory. Second, based on the intrinsic limitations of these explicit personality measures, we review a key set of results showing effects of MM on implicit, as well as explicit, self-representations. Although the research on MM and personality is still in its infancy, it appears that this form of meditative practice may notably shape individuals’ personality and self-concept toward more healthy profiles.

## INTRODUCTION

Meditation originated several centuries BCE in Hinduism and Buddhism healing and spiritual traditions. Since then, various forms of meditation are followed to cultivate well-being and cognitive/emotional balance (Wynne, 2007; Lutz et al., 2008; Fabbro, 2010). Over the past 30 years, an important form of meditation has taken hold in the West, in both clinical and non-clinical contexts, namely mindfulness meditation (MM; Kabat-Zinn, 1982, 1990, 2003). Mindfulness meditation relies upon techniques of mental training that suggest that non-judgmental awareness of here-and-now mental or somatosensory experience positively influences accurateness of perception and acceptance of one’s own life experiences. The mindful practitioner thus amalgamates a focused attention component with a non-judgmental attitude of openness and receptivity when trying to intentionally pay attention, and non-reactively monitor, the content of present-moment experience (Brown and Ryan, 2003; Lutz et al., 2008).

The growing interest in MM is probably due to its well-documented beneficial effects on physical and psychological health, which have been obtained in different clinical contexts on individuals of various age ranges and with slightly different formalizations of mindfulness-based therapies (Baer, 2003, 2010; Didonna, 2009). Moreover, another important dimension of MM is its potential in promoting personal development such as equanimity, self-compassion, and perspective taking (Neff, 2003; Baer, 2010; Birnie et al., 2010; Hölzel et al., 2011) and in undermining self-critical tendencies (Hayes et al., 2004; Segal et al., 2004).

Together with the body of knowledge pointing to the health benefits of MM, another research line has aimed at identifying the mechanisms of action through which MM exerts its effects. Thus, the role of a series of interrelated components such as attentional control, emotion regulation, body awareness, and change in perspective on the self has been recognized (Hölzel et al., 2011). Nevertheless, while several studies have focused on the relation between MM and enhancement of attention and emotion regulation skills (e.g., Jha et al., 2007; Lutz et al., 2008, 2014; Malinowski, 2013; for a recent review see Lippelt et al., 2014), relatively little empirical research has investigated whether MM promotes changes in the perspective on the self and, more generally, in personality traits (Hölzel et al., 2011; Campanella et al., 2014). However, a central tenet of MM is that the practitioner learns to experience the transient nature of all mental phenomena including the sense of self, gaining distance (detaching) from identification with a static sense of self that, instead, would be experienced as an event. Experiencing the transitory nature of the sense of self has been suggested to lead to the “deconstruction of the self” (Epstein, 1988; see in Hölzel et al., 2011), which presumably may promote modifications in personality. Moreover, it is also generally assumed that the practice of MM aims at developing new ways to experience and face life events (Kabat-Zinn, 1990; van den Hurk et al., 2011) and this may also lead to changes in personality. From this perspective, the empirical study of the relation between MM and personality is of some importance as it is reasonable to expect modifications in personality traits as due to MM practice.

## MINDFULNESS MEDITATION AND EXPLICIT MEASURES OF PERSONALITY AND SELF-CONCEPT CHANGES

One of the first studies documenting a change in individuals’ self-concept as due to MM has been that of Emavardhana and Tori (1997; see also Nystul and Garde, 1977 and Turnbull and Norris, 1982 for two older studies), who reported higher self-acceptance, increases in overall self-esteem and a more positive self-representation (measured with the Tennessee Self-Concept Scale; Roid and Fitts, 1988), in two large groups (overall *n* = 438) of 18-years old participants attending a 1-week mindfulness retreat (i.e., vipassana meditation) vs. a control group of young participants (*n* = 281).

More recently, several studies have further investigated the relationship between MM and personality relying on well-known personality inventories. Many of these studies have tried to link dispositional (trait) mindfulness (globally reflecting the disposition to persist in mindful states over time irrespective of meditation practice; Brown et al., 2007), rather than MM practice, with personality traits using the Five-Factor model of personality (e.g., Costa and McCrae, 1992). For example, Thompson and Waltz (2007) employed the Mindful Attention Awareness Scale (MAAS, Brown and Ryan, 2003), a 15-item mindfulness scale regarding levels of attention and awareness in daily life, finding that greater mindfulness scores were negatively related with the neuroticism trait (propensity toward anxiety, worrying, moodiness, impulsiveness, and self-criticism) but positively related with the agreeableness (the tendency to be trustworthy and altruistic) and conscientiousness (the inclination to be efficient, organized and to show high self-discipline) traits. Similarly, Barnhofer et al. (2011) have shown that dispositional mindfulness [measured in this study with the Five-Facet Mindfulness Questionnaire (FFMQ), Baer et al., 2006] moderated the relation between depressive symptoms and neuroticism, which were positively related only at low or medium, but not at high levels of dispositional mindfulness. Other studies have documented negative correlations between dispositional mindfulness or self-compassion and neuroticism (Brown and Ryan, 2003; Neff et al., 2007; Ortner et al., 2007; Zabelina et al., 2011), while in one of these studies it was also found that state mindfulness (the moment-to-moment awareness of one’s experience; a state that can be enhanced by practices such as brief MM exercises) facilitated creative elaboration among individuals with high levels of neuroticism. This was proposed to occur via a role of mindfulness in reducing tendencies toward self-criticism and behavioral inhibition (Zabelina et al., 2011). These and other data linking mindfulness and personality traits have recently been subjected to meta-analysis (Giluk, 2009) involving 29 studies addressing the relationship between dispositional mindfulness (e.g., trait MAAS, FFMQ) and the Big-Five personality traits (conscientiousness, agreeableness, extraversion, openness-to-experience, and neuroticism). Overall, this meta-analysis confirmed that trait mindfulness negatively correlates with neuroticism but positively with conscientiousness.

Although valuable, these studies interested in the relationship between everyday mindfulness and personality leave open the question of the impact of regular MM practice on mindfulness and personality variables, for instance during an 8-week intervention or in expert practitioners. Of importance, a recent study by van den Hurk et al. (2011) has recently made an important step toward understanding the contribution of MM practice. In this cross-sectional study the authors compared the personality profiles (using the NEO-Five-Factor Inventory; Costa and McCrae, 1992) of two groups of healthy participants (age range, 27–75 years) with (*n* = 35; mean meditation experience: 13.2 years, 3.0 h a week) and without (*n* = 35) MM experience. Meditators showed higher openness-to-experience scores (reflecting dispositional curiosity, creativity) than non-meditators but lower conscientiousness scores than the latter. Moreover, the practice of MM was negatively related to neuroticism and positively related to openness-to-experience and extraversion (the tendency to experience positive emotions and being sociable). Although the cross-sectional nature of the study may limit the interpretation of the results, the authors concluded that MM practice is associated with positive affect and increased levels of curiosity and openness-to-experience. Furthermore, it helps to reduce moodiness and worrying, also leading to a reduced focus on achievements (related to the conscientiousness trait).

Another well-known personality inventory refers to the psychobiological model of Temperament and Character (TCI; Cloninger et al., 1993, 1994). This model combines the posited neurobiological and genetic bases of personality (four temperamental traits: Novelty Seeking, Persistence, Reward Dependency, and Harm Avoidance) with their interaction with life experiences (character). The character consists of three dimensions measuring the maturity of the self at the levels intrapersonal (Self-Directedness that maps on concepts such as self-esteem and self-efficacy), interpersonal (Cooperativeness expressing the capacity to be empathic, tolerant, compassionate), and transpersonal (Self-Transcendence measuring the tendency toward spirituality and creativeness). Thus, the character refers to one’s own self-evaluation and is responsible for efficient behavioral self-regulation; indeed, character profiles are useful to diagnose personality disorders, in that individuals with immature character (e.g., with low Self-Directedness and Cooperativeness) are at higher risk of developing personality disorders than individuals with better character maturity (Svrakic et al., 1993, 2002).

Only a few studies have used the TCI to investigate the relationship between dispositional mindfulness or MM practice and personality traits. Haimerl and Valentine (2001) compared cross-sectionally the TCI character profiles of three groups of Buddhist meditators with varying levels of meditation experience (28 naïve individuals; 58 beginners with less than 2 years of practice; and 73 experts with more than 2 years of experience). Indicative of greater overall self-maturity, expert meditators obtained higher scores in all three aspects of the character profile compared to naïve subjects and they also scored higher than beginners on the cooperativeness trait. Moreover, beginners scored higher than naïve individuals on the self-transcendence scale. This study thus showed that progresses in meditation experience led to positive growth in the character components of personality (see Smalley et al., 2009 for similar findings obtained considering dispositional mindfulness rather than MM practice).

Similarly to the conclusions that can be drawn from the studies using the Five-Factor model of personality, the correlative and cross-sectional nature of these previous studies considering the TCI may limit the possibility of linking MM with the changes in the perspective on the self. Nevertheless, a recent longitudinal study by Campanella et al. (2014) was able to test more directly the effects of MM practice on individuals’ TCI personality profiles. This was done in three groups of meditation-naïve healthy subjects (overall *n* = 41, age range: 21–58 years) who participated in three replicates of an 8-week MM course inspired by the mindfulness-based stress reduction. In this study, there was also a control group of healthy individuals (*n* = 15, age range: 26–69 years) not involved in any meditation training. Notably, the authors reported increased scores in all three character scales after vs. before the MM course in two of the three meditation groups (groups 1 and 3 in Campanella et al., 2014, Figure 1). In the remaining meditation group (group 2 in Campanella et al., 2014, Figure 1) and in the control group the TCI profiles remained unaltered across the two testing sessions. Remarkably, it was found that the individuals in the two meditation groups who showed an increment in the character scores had meditated more frequently than those in the meditation group who did not show any character change during the course (4–5 vs. 2–3 days per week, respectively; see Figure 2A in Campanella et al., 2014).

Overall, the reviewed studies above suggest that MM may indeed promote positive changes in individuals’ self-concept and personality. This may help further characterizing the change in the perspective on the self as an important mechanism of action through which MM exerts its beneficial health effects. In particular, this change in perspective could be supported by a mindfulness-related increased ability to start experiencing the sense of self as a transitory event rather than as a constant and unchanging entity (Hölzel et al., 2011; see Introduction). The detachment from identification with a static sense of self may provide MM practitioners with a better capacity for objectivity about their own internal experience that, in turn, could help them experience more authentic ways of being and reduce psychological suffering (Shapiro et al., 2006; Hölzel et al., 2011).

Tellingly, recent functional imaging studies have identified the putative neurofunctional signatures of the change in the perspective on the self brought about by MM. In particular, it has been claimed that during MM states detachment from identification with a static sense of self associates with a diminished self-referential, narrative/autobiographical, processing paired with enhanced present-based, experiential processing of the self (Farb et al., 2007). In the brain, this has been shown to reflect in decreased activity in self-referential cortical midline structures (e.g., medial prefrontal cortex; see Northoff and Bermpohl, 2004; Northoff et al., 2006) and enhanced activity in lateral structures such as the insula and the somatosensory cortex associated more with momentary interoceptive and exteroceptive self-awareness (see Hölzel et al., 2011 for a discussion of other relevant neuroimaging studies; see also Tomasino et al., 2013).

## MINDFULNESS MEDITATION AND IMPLICIT MEASURES OF PERSONALITY AND SELF-CONCEPT CHANGES

People have two sources of self-evaluative tendencies. The first relies on propositional processes of intentional reasoning that shapes individuals’ explicit attitudes through well-articulated beliefs and motivations. By contrast, the second source roots in associative and automatic processes in which intuitive feelings and evaluations, that one could or could not be aware of, shape individuals’ implicit attitudes (Gawronski and Bodenhausen, 2006; Jordan et al., 2007). While explicit attitudes can be measured through self-report questionnaires (e.g., TCI), implicit attitudes can be inferred from individuals’ performance on reaction times measures such as the Implicit Association Test (IAT). The IAT is one of the most common tests used to compute the strength of automatic concept-attribute associations that could underlie specific aspects of personality (Greenwald and Farnham, 2000; Schnabel et al., 2008). Relative to self-report measures, implicit tests do not require the intent to self-evaluate on the part of the respondent and are thus more difficult to fake or to control (Greenwald and Farnham, 2000; Crescentini et al., 2014a). Of importance, experiencing psychological conflicts between intuitive feelings and more reflective evaluations is common in daily life decisions, with such conflicts also affecting more personal spheres, such as self-attitudes and self-representations (Emmons and King, 1988; Greenwald and Banaji, 1995; Gawronski and Bodenhausen, 2006). Notably, while concordance between implicit and explicit self-representations is important for psychological health, incongruities between the two forms of self-evaluation have been put into relation with different forms of psychological distress. For example, unhealthy forms of perfectionism may occur when one shows high implicit self-esteem but low explicit self-esteem (Zeigler-Hill and Terry, 2007; see also Bosson et al., 2003; Briñol et al., 2006; Schröder-Abé et al., 2007a,b).

On the basis of these premises, it seems important to investigate the impact of MM upon changes in the perspective on the self also at the implicit level of self-representation. This appears critical for at least two reasons. First, linking mindfulness with implicit self-concept, namely a specific type of implicit cognition, may help to “cross-validate” findings from self-report measures of personality. Explicit measures may indeed be susceptible to desirable responding and are clearly subjective in nature (Schwarz, 1999; Jordan et al., 2007). Second, these studies could test whether mindful awareness promotes a coherent self in which implicit and explicit self-representations become better integrated with each other. Despite intuition and implicit cognition are likely important aspects of mindfulness, as this practice is believed to foster self-insights and greater acceptance of one’s own internal states, we should note that most of the studies on the impact of everyday mindfulness or MM practice on personality and self-concept have only considered explicit self-report measures.

Nevertheless, a few relevant exceptions exist, being represented by studies focusing on the impact of mindfulness on psychological dimensions such as implicit affective states, self-esteem, and motivation (Brown and Ryan, 2003; Levesque and Brown, 2007; Koole et al., 2009; Sauer et al., 2011; see Hutcherson et al., 2008 and Strick et al., 2012 for similar issues in the context of Loving-Kindness meditation and Zen meditation). Overall, these studies have been particularly interested in the putative effects of dispositional mindfulness or transient state mindfulness on implicit and explicit self-representations and/or their potential concordance. Thus, in their pioneering study, Brown and Ryan (2003, Study 3) measured individuals’ emotional well-being using both self-report measures (explicit level) and an IAT (implicit level). They found that MAAS scores predicted concordance between implicit and explicit affect. In particular, there was a closer relation between explicit and implicit affective experience in meditation naïve individuals (*n* = 78) with high versus low MAAS scores. Another important study has recently extended these findings to self-esteem (Koole et al., 2009). In particular, it was shown that brief MM exercises, carried out by young naïve participants (overall *n* = 188), led to greater congruence between explicit and implicit measures of self-esteem specifically when they were executed before, rather than after, completing the two types of self-esteem.

Notably, these studies interested in dispositional or state mindfulness left unaddressed the issue of whether regular MM practice, for instance during an 8-week mindfulness-based intervention, directly affects implicit as well as explicit self-concepts. This is an important issue that was addressed by our research group in a recent study (Crescentini et al., 2014b). In particular, we assessed the changes in explicit (e.g., Self-Transcendence in the TCI) and implicit [using an IAT for religiousness/spirituality (RS); Crescentini et al., 2014a] RS self-representations in meditation-naïve individuals participating in an 8-week MM program. Remarkably, we found that MM led to widespread increases in explicit RS and to more circumscribed increases in implicit RS occurring in the individuals with low pre-existing implicit RS (i.e., before the MM training). Moreover, the two RS measures globally tended to increase congruently after vs. before the training.

Generally, the reviewed studies suggest that MM may have an impact on implicit self-concepts. This is important if one considers that implicit attitudes may be more difficult to transform than explicit attitudes; while the latter may represent recently acquired self-representations coexisting with the former, implicit attitudes could reflect more stable and older evaluative representations that have their origins in long-term personal experiences (Wilson et al., 2000; Gawronski and Bodenhausen, 2006). Development of mindful awareness thus appears able to impact responding at an automatic level and it could therefore be involved in gradually transforming patterns of automatic and habitual reacting and self-evaluation (Chambers et al., 2008). More generally, the reviewed data suggest that this practice fulfills important self-regulatory functions, for instance by letting intuitive self-attitudes to be more easily attuned and integrated into explicit self-attitudes, thus contributing to a more coherent self-image (Koole et al., 2009; Crescentini et al., 2014b).

## CONCLUSION

The aim of this study was to review findings of researches investigating the relationship between MM and changes in personality and in the perspective on the self. Most of these studies addressed changes occurring at an explicit level using self-report measures of personality and self-concept changes. However, a few studies also focused on changes occurring at a deeper, implicit level. Although the research on MM and personality is still in its infancy, warranting further investigations on both levels of explicit and implicit self-concepts, the reviewed studies suggest that, operating on aspects such as sense of responsibility, authenticity, compassion, and self-acceptance, this form of mental training may significantly shape individuals’ personality toward a more coherent and healthy sense of self and identity.

## Conflict of Interest Statement

The Guest Associate Editor Barbara Tomasino declares that, despite being affiliated to the same institution as the authors, the review process was handled objectively and no conflict of interest exists. The authors declare that the research was conducted in the absence of any commercial or financial relationships that could be construed as a potential conflict of interest.
